# Influence of Steel and Polypropylene Fibers on Flexural Strength and Fracture Properties of Ambient-Cured Geopolymer Concrete

**DOI:** 10.3390/polym18070873

**Published:** 2026-04-02

**Authors:** Mustafa Oguz, Süleyman Özen, Şemsi Yazıcı, Ali Mardani

**Affiliations:** 1 Department of Civil Engineering, Faculty of Engineering, Ege University, Izmir 35100, Türkiye; mustafaoguz@gmail.com (M.O.); semsi.yazici@ege.edu.tr (Ş.Y.); 2 Department of Civil Engineering, Faculty of Engineering and Natural Sciences, Bursa Technical University, Bursa 16059, Türkiye; suleyman.ozen@btu.edu.tr; 3 Department of Civil Engineering, Faculty of Engineering, Bursa Uludag University, Bursa 16059, Türkiye

**Keywords:** geopolymer concrete, fiber reinforcement, sustainable construction, brittleness, toughness, mechanical properties

## Abstract

The environmental urgency of reducing Portland cement consumption has driven research into geopolymer concrete (GPC) as a sustainable alternative. However, its inherent brittleness limits structural applications. This study addresses this critical challenge by investigating the efficacy of steel fibers (SF) and polypropylene fibers (PP) in enhancing the mechanical properties of slag-based GPC. Thirteen mixtures, including a control, were designed with varying fiber types, lengths (35/60 mm for SF, 40/60 mm for PP) and dosages (25–75 kg/m^3^ for SF, 3–9 kg/m^3^ for PP). Comprehensive tests evaluated workability, flexural/compressive strength, and toughness. Results demonstrated that while both fibers reduced workability (PP > SF), they significantly improved ductility, with SFs increasing toughness by 6–15 times. A key finding was the time-dependent performance: SF enhanced early-age flexural strength by up to 38%, though this benefit declined at 28 days for most mixes under ambient curing. PP fibers reduced flexural strength by 25–40% at 28 days. Compressively, SF increased strength by up to 60%, while PP led to reductions up to 27%. The study conclusively establishes SF’s superiority due to its superior bonding and crack-bridging capabilities, providing essential insights for designing durable fiber-reinforced GPC. This research directly contributes to advancing sustainable construction materials by overcoming a fundamental limitation of geopolymers.

## 1. Introduction

Concrete is the second most consumed material globally, surpassed only by water, underscoring its indispensable role in the rapid development of modern infrastructure [1]. However, the environmental footprint associated with conventional concrete production, particularly that of Ordinary Portland Cement (OPC), is substantial and a growing global concern [1,2]. Cement manufacturing is a major contributor to environmental degradation, characterized by high energy consumption and raw material extraction. The production of a tonne of OPC requires approximately 1.5 tonnes of raw materials and releases a comparable mass of carbon dioxide (CO_2_) into the atmosphere [1]. Consequently, the cement industry alone accounts for an estimated 8–10% of CO_2_ emissions, presenting a significant sustainability challenge for the construction sector and driving an urgent demand for alternative binders [2,3,4,5].

Geopolymer concrete (GPC) has been widely characterized as a transformative construction material with the potential to reduce carbon emissions by 16% to as much as 99% compared with traditional Ordinary Portland Cement (OPC) concrete [6,7,8]. This environmental advantage largely stems from the elimination of energy-intensive limestone calcination, a key process in OPC production that accounts for approximately 5–8% of global anthropogenic CO_2_ emissions [9,10].

Geopolymer or alkali-activated binders are therefore increasingly investigated as viable substitutes for OPC, as they can be produced from aluminosilicate precursors—often industrial by-products such as fly ash or blast furnace slag—activated using alkaline solutions. This approach can reduce reliance on clinker production while simultaneously valorizing industrial wastes and lowering associated emissions [11,12,13].

Comparative assessments consistently report substantial reductions in carbon footprint for geopolymer and alkali-activated systems relative to OPC-based materials, with many studies indicating reductions of approximately 40–60% when explicit life-cycle percentages are reported. However, these benefits exhibit considerable variability and may involve cost trade-offs, largely influenced by supply-chain factors and formulation choices, particularly the type and quantity of alkaline activators and reinforcement or composite constituents [11,13,14,15].

Despite these advantages, GPC shares a critical limitation with its OPC counterpart: inherent brittleness [16,17]. GPCs typically exhibit quasi-brittle behavior, characterized by a relatively low tensile and flexural strength compared to their compressive strength, often resulting in sudden, catastrophic failure. This brittleness, coupled with a lower elastic modulus than OPC, makes GPC susceptible to cracking and limits its application in structural elements subjected to tensile or flexural stresses [18,19]. To mitigate this drawback and enhance the deformation capacity, toughness, and crack resistance of GPC, the incorporation of discrete fibers has been established as an effective strategy. Fibers act to bridge micro-cracks, control volumetric changes during shrinkage and ultimately transition the material’s failure mode from brittle to more ductile. The performance of these fiber-reinforced composites is predominantly governed by the properties of the fiber–matrix interface [17,20,21].

Recent developments highlight that fiber-reinforced materials are not only essential for new constructions but also offer significant potential for the strengthening and rehabilitation of existing reinforced concrete structures damaged by factors like fire or earthquakes. Fiber-reinforced polymers (FRPs) have gained widespread attention due to their high tensile strength, lightweight nature, and corrosion resistance.

One critical advantage of these materials is their resistance to elevated temperatures. Studies indicate that while concrete loses approximately 40% of its compressive strength when exposed to temperatures like 650 °C, the application of basalt fiber reinforcing bars and carbon fiber-reinforced polymer ropes using the near surface mounted technique can enhance the shear capacity of heat-damaged beams by 37% to 95% [22].

Furthermore, innovative configurations, such as closed-form retrofitting using FRP ropes embedded in surface grooves, have been shown to significantly improve the performance of non-rectangular sections like T-beams. These methods effectively delay debonding and can increase torsional strength by up to 25% while substantially enhancing ductility at failure. Including these current trends in fiber applications strengthens the understanding of geopolymer concrete’s role in modern structural engineering [23].

Among various options, polypropylene (PP) fibers are widely used for reinforcing cementitious and geopolymer composites due to their cost-effectiveness and beneficial properties [19,24]. Their hydrophobic nature reduces interaction with the aqueous geopolymer binders, facilitating easier mixing [1]. PP fibers are renowned for significantly improving the post-cracking behavior of the matrix by slowing crack propagation, thereby enhancing overall toughness at a relatively low cost. Studies indicate that incorporating PP fiber at volumes up to 2.0% can improve flexural properties, with optimal contents around 0.5% or 1% often enhancing compressive strength, flexural strength, and overall geopolymer performance [19,20,25,26].

However, the addition of PP fibers also introduces challenges, primarily a significant reduction in the fluidity and workability of fresh geopolymer mixes. This is attributed to their low density, which leads to a higher number of fibers per unit volume, increasing fiber–matrix interactions [1,3,26,27]. Higher volume fractions (e.g., >1.75% for UHPGPC) can result in harsh mixes, and excessive fiber content can cause balling and poor dispersion, compromising mechanical properties [16,17,28]. Furthermore, some studies report that higher percentages of PP fibers can adversely affect strength, particularly compressive strength with reductions of 11.3% at 0.5% and 44.3% at 4% fiber content reported at 56 days [17,18,25,26].

In contrast, steel fibers (SFs) are renowned for their ability to impart superior mechanical properties to GPC [18]. Their hydrophilic nature and rough surface promote strong interfacial bonding with the geopolymer matrix, leading to remarkable improvements in energy absorption, flexural strength, compressive strength and splitting tensile strength [17,18,27,29]. The compressive strength of steel fiber-reinforced geopolymer composites has been shown to peak at around a 2.5% volume fraction, demonstrating improvements of over 13% compared to control mixes [2]. SF additions also contribute to reducing drying shrinkage and mitigating damage from elevated temperatures. Optimal dosages typically range from 1% to 3% to significantly enhance compressive and flexural behavior [27]. Similar to PP fibers, the incorporation of SF leads to a considerable decrease in workability [2,27].

Fiber-reinforced geopolymer composites are commonly analyzed using constitutive damage–plasticity models and micromechanical homogenization approaches to predict their elastic response, damage evolution, and fracture behavior. The microplane damage–plasticity model, implemented in software such as ANSYS, has been used to simulate geopolymers reinforced with natural fibers (e.g., sisal), where fibers are treated as inclusions within the geopolymer matrix, allowing accurate representation of hardening and post-peak softening behavior [30]. For wood-fiber-reinforced geopolymers, the exponential Drucker–Prager yield criterion coupled with the Concrete Damage Plasticity (CDP) model in Abaqus is often applied to capture elastic response and progressive degradation under compressive and flexural loading [31]. At the micromechanical scale, the Mori–Tanaka homogenization method, based on Eshelby’s inclusion theory, predicts effective elastic properties such as Young’s modulus for basalt-fiber-reinforced geopolymer concrete [32]. Fracture performance is frequently evaluated using the double-K fracture model, derived from load–CMOD curves, to estimate crack initiation and unstable propagation [32].

While numerous studies have examined the effects of fiber reinforcement in cementitious and geopolymer composites, including steel, polypropylene, and hybrid fiber systems, the reported results vary depending on binder composition, fiber geometry, and dosage. In many cases, studies have focused on individual fiber types or limited mechanical properties, which makes direct comparisons within the same geopolymer system more difficult.

In particular, comparative experimental data evaluating the influence of steel and polypropylene fibers on both fresh-state behavior and key mechanical properties of slag-based geopolymer concretes remain relatively limited. Providing such data can help clarify the practical implications of fiber type and dosage in these materials.

Accordingly, this study experimentally investigates the effects of different volume fractions of steel and polypropylene fibers on the fresh properties, compressive strength, and flexural strength of slag-based geopolymer concrete, aiming to provide additional experimental evidence to support the understanding and practical application of fiber-reinforced geopolymer concretes.

## 2. Materials and Methods

### 2.1. Materials

**Figure 1:**
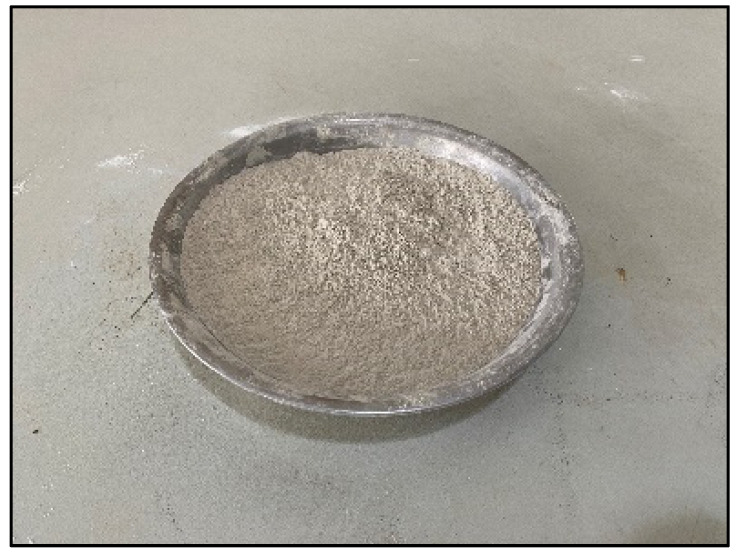
Ground granulated blast furnace slag.

In the experimental study, ground granulated blast furnace slag (GGBFS), obtained from Kent Beton, Izmir, Türkiye, was used as the binder (Figure 1).

**Figure 2:**
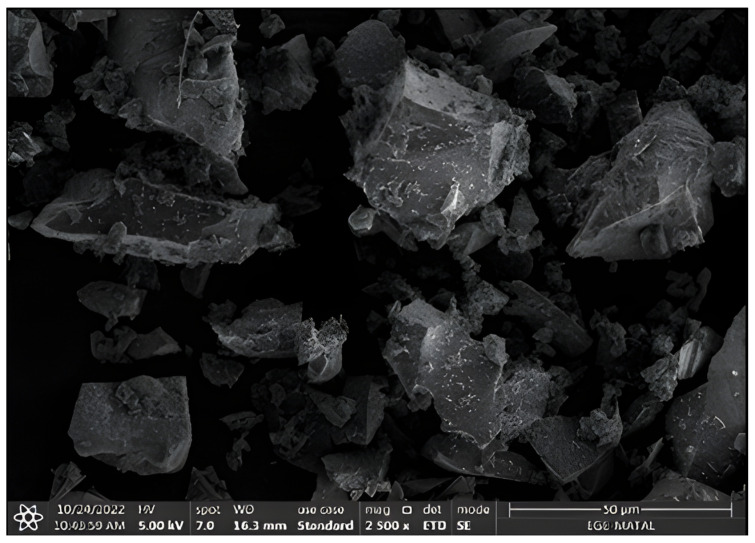
SEM image of the blast furnace slag used in the experimental study.

The specific gravity of the GGBFS is 2.79, and its specific surface area is 2370 g/cm^2^. Its chemical composition is given in Table 1, while the scanning electron microscope (SEM) image is shown in Figure 2.

The activator solution for geopolymer mixes was prepared using sodium silicate (Na_2_SiO_3_) and sodium hydroxide (NaOH), both procured from local suppliers in Izmir, Türkiye. Their physical and chemical properties are given in Table 2 and Table 3.

Crushed limestone aggregates of three size ranges (0–5 mm, 5–15 mm, and 15–25 mm), procured from local suppliers in Izmir, Türkiye, were used. The physical properties of the aggregates, determined according to TS EN 1097-6 [33], are presented in Table 4. The sieve analyses according to TS EN 933-1 [34] are provided in Table 5.

**Figure 3:**
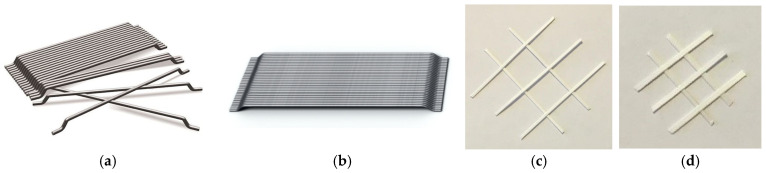
Fibers used in geopolymer mixtures. (**a**) 60 mm steel fiber, (**b**) 35 mm steel fiber, (**c**) 60 mm polypropylene fiber, (**d**) 40 mm polypropylene fiber.

Steel fibers (BEKAERT, Izmir, Türkiye) and polypropylene fibers (KORDSA, Izmir, Türkiye) were used in the mixtures (Figure 3). Their main properties are summarized in Table 6. The steel fibers are hooked-end fibers with lengths of 3.5 and 6 cm, while the PP cut fibers have varying diameters and lengths of 4 and 6 cm.

### 2.2. Test Method and Mix Proportions

The alkaline activator solution for all geopolymer mixtures was prepared by mixing sodium silicate (Na_2_SiO_3_) with sodium hydroxide (NaOH) to achieve a fixed silicate modulus (Ms = SiO_2_/Na_2_O ratio) of 1.40. A constant binder content, consisting solely of ground granulated blast furnace slag (GGBFS), was maintained across all mixes. The water-to-binder (W/B) ratio, calculated by accounting for both the added mix water and the water present in the sodium silicate solution, was held constant at 0.55. In fiber-reinforced mixtures, the volume of incorporated fibers directly replaced an equivalent volume of aggregate.

**Figure 4:**
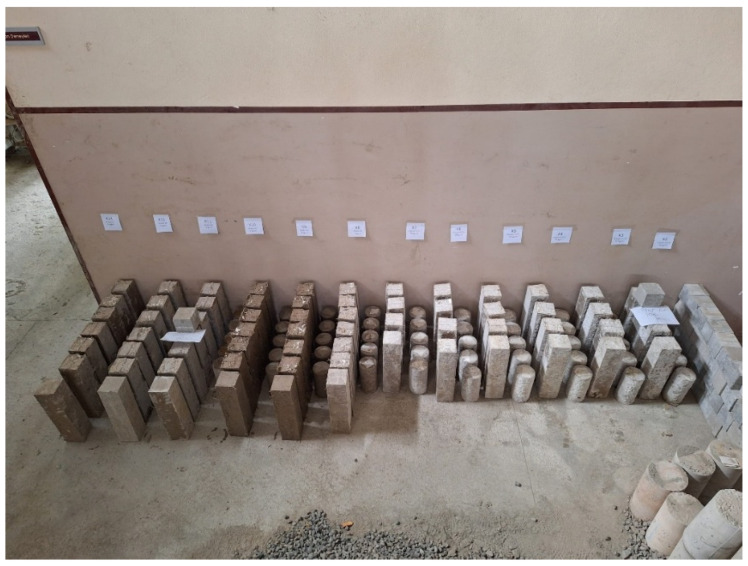
Specimens produced in the laboratory.

Two distinct fiber types, steel (SF) and polypropylene (PP), were investigated, each at two different lengths and three dosage levels. This experimental plan resulted in a total of 13 distinct mixtures, including a control mix without fibers. For each experiment, three specimens were prepared to ensure the reliability of the results and allow statistical evaluation. If a high standard deviation was observed among the specimens within the same mixture, the experiment was repeated. Steel and PP fibers were incorporated at volumetric fractions of 0.3%, 0.6%, and 0.9%, corresponding to 25, 50, and 75 kg/m^3^ for steel fibers and 3, 6, and 9 kg/m^3^ for PP fibers, respectively. All prepared specimens were demoulded after 24 h and subsequently stored at constant laboratory temperature 25 °C until testing at 7 and 28 days, without employing any additional curing measures. The detailed mix proportions for all 13 mixtures are provided in Table 7, and a photograph of the laboratory-produced specimens is shown in Figure 4.

**Figure 5:**
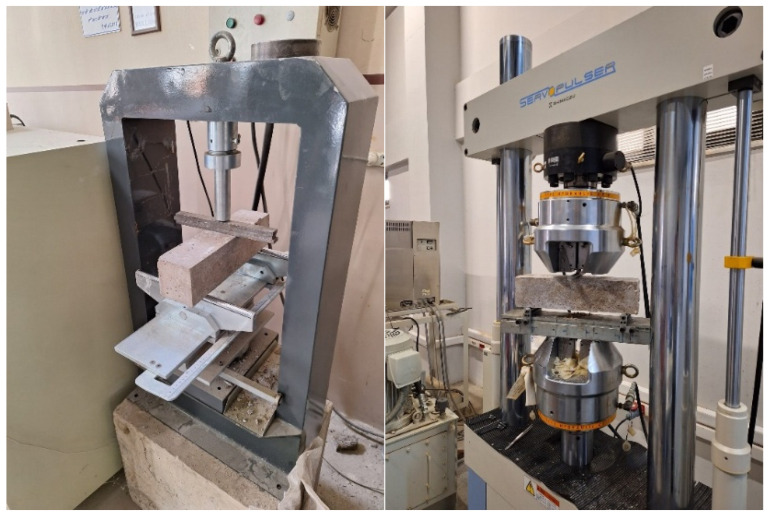
Three-point bending test setups.

The workability of the fresh geopolymer concrete was evaluated using the slump test, conducted in compliance with the standard TS EN 12350-2 [35]. The mechanical characterization of hardened concrete involved compressive and flexural strength tests. Flexural strength was determined using three-point bending tests on prismatic specimens measuring 100 × 100 × 400 mm, following the procedures specified in TS EN 12390-5 [36]. The span length was set to 300 mm. The specimens were loaded at a rate of 10 kg/s, and displacement was measured using a linear variable differential transformer (LVDT, UTEST, Ankara, Türkiye). Experiments were performed on nominally unnotched specimens. Specimens, tested at 7 days, were loaded solely to determine the maximum flexural strength value, without recording deformation data. In contrast, the 28-day tests were performed under displacement-controlled conditions, enabling the simultaneous capture of load corresponding mid-span deflection to construct detailed load–displacement curves. The experimental setup for the flexural tests is presented in Figure 5. Flexural strength was calculated using the following equation, where *σ* is the flexural strength (MPa), *P* is the maximum applied load (N), *L* is the beam span (mm), and *H* is the beam height (mm).

$$\sigma =\frac{3P\times L}{{2H}^{3}}$$

Flexural toughness was calculated according to ASTM C1609 [37] using the trapezoidal method, where *T* represents flexural toughness, *P* the applied load, and *δ* the corresponding deflection.

$$T=\Sigma \frac{\left(P_{i}+P_{i+1}\right)}{2}\left(\delta_{i+1}+\delta_{i}\right)$$

Compressive strength was conducted on 100 mm cube specimens at 7 and 28 days of age, strictly adhering to the guidelines of TS EN 12390-3 [38]. For each mixture and test age, three specimens were tested, and the reported result represents the arithmetic mean of the measured values.

## 3. Results and Discussion

### 3.1. Slump-Test

**Figure 6:**
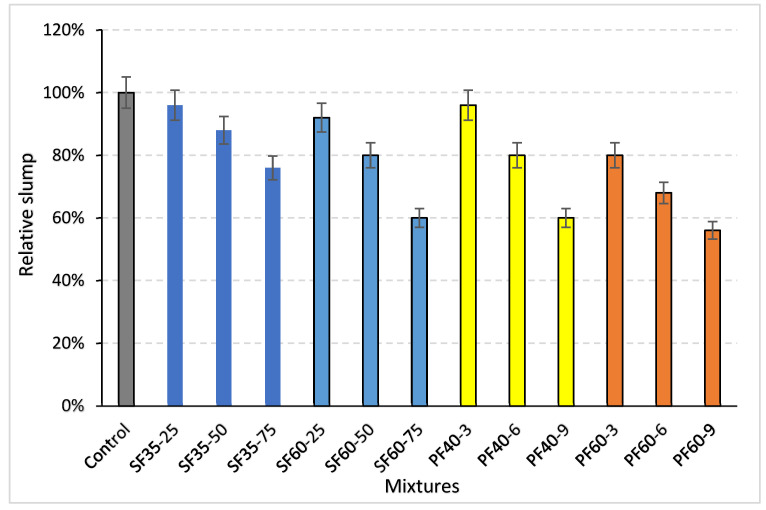
Relative slump values of the mixtures. Different colors represent different mixture groups: gray indicates the control mixture, blue tones represent steel fiber (SF) mixtures, yellow tones represent polypropylene fiber mixtures with 40 mm length (PF40), and orange tones represent polypropylene fiber mixtures with 60 mm length (PF60).

The measured slump values for all geopolymer concrete mixtures are summarized in Table 8. The relative slump of each mixture, expressed as a percentage of the control mix value, is presented in Figure 6.

The incorporation of fibers, irrespective of type, resulted in a reduction in the workability of the fresh geopolymer concrete, as evidenced by decreasing slump values. Although steel and PP fibers were introduced at approximately equivalent volumetric proportions, PP fibers exerted a more detrimental effect on the consistency of the mixtures. A clear trend was observed where an increase in either fiber length or dosage level induced a progressive decline in slump. The maximum reduction in slump reached 40% for steel fiber mixtures and approximately 45% for those containing PP fiber, relative to the fiber-free control mix.

This observed reduction in workability aligns with findings from previous studies. Ranjbar et al. [17] attributed similar slump losses with higher fiber contents to an increase in the mixture’s shear resistance. The results are also consistent with reports by Aisheh et al. [1] and Wang et al. [18], who documented diminished workability and flow diameter with escalating fiber content, as well as other studies on fiber-reinforced concrete systems [39]. Wang et al. [18] further suggested that the pronounced reduction in fluidity in PP-fiber mixtures is primarily due to fiber entanglement during the mixing process.

The mechanism behind the more significant workability loss with PP fibers can be explained by their physical properties. Despite their hydrophobic nature, which minimizes interaction with the aqueous phase of the geopolymer binder, the low specific gravity of PP fibers results in a significantly higher number of individual fibers per unit volume compared to steel fibers at an equivalent mass. This substantially increases the total surface area and the number of fiber–matrix interactions, thereby elevating the internal resistance to flow and impairing workability more severely [1].

Furthermore, increasing the fiber content enhanced the overall viscosity of the mixture, which promotes internal friction and the tendency for fibers to clump or ball together. The negative effect on workability was exacerbated by longer fiber lengths, which increase the potential for entanglement and interaction within the matrix [40,41]. While both fiber length and content were determined to adversely affect slump, the analysis indicates that fiber content was the predominant factor governing the reduction in workability.

### 3.2. Flexural Strength

**Figure 7:**
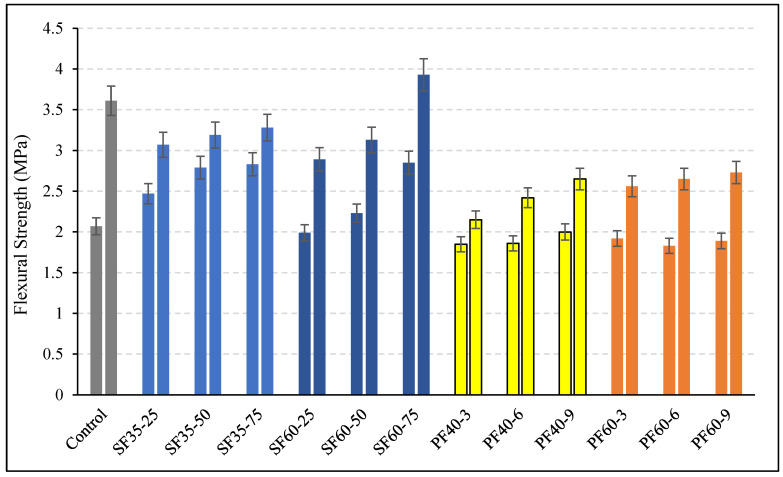
The 7- and 28-day flexural strengths of the mixtures. Different colors represent different mixture groups: gray indicates the control mixture, blue tones represent steel fiber (SF) mixtures, yellow tones represent polypropylene fiber mixtures with 40 mm length (PF40), and orange tones represent polypropylene fiber mixtures with 60 mm length (PF60).

The flexural strength results (Figure 7) present a nuanced and time-dependent picture of fiber reinforcement in geopolymer concrete. The most striking finding is the divergent behavior between early and later ages, particularly for steel fiber-reinforced mixes, which contrasts with typical performance in Ordinary Portland Cement (OPC) systems.

The significant enhancement in 7-day flexural strength with steel fiber incorporation aligns with established mechanisms reported in the literature [42,43,44]. The observed improvements, reaching up to 38% for the SF60-75 mixture, can be primarily attributed to the effective crack-bridging effect of steel fibers, which helps distribute localized stresses and delays the propagation of micro-cracks [16,45,46]. Specimens with 35 mm steel fibers performed better than the control, with strength gains ranging between 20% and 37%, showing a consistent improvement with increasing fiber content. The hydrophilic nature and rough surface topography of steel fibers promote a strong initial interfacial bond with the geopolymer matrix, allowing for efficient stress transfer at early ages [17].

However, a drastic diminution of this positive effect was observed at 28 days for most steel fiber mixes, constituting a critical finding of this study. Contrary to common literature findings where steel fibers typically enhance mechanical properties at maturity [16], the experimental data indicate that only the mix with the highest dosage of long fibers (SF60-75) marginally exceeded the control mix strength, showing a 9% increase. All other steel fiber mixtures performed worse than the control, with reductions of up to 20%.

The most plausible explanation for this strength regression lies in the interplay between time-dependent matrix behavior and the fiber–matrix interface under ambient curing conditions. The potentially incomplete geopolymerization reaction at 25 °C may have resulted in a weaker matrix microstructure that is more susceptible to damage under sustained loading. More critically, ongoing autogenous and drying shrinkage in the geopolymer matrix can induce progressive tensile stresses, leading to a degradation of the fiber–matrix bond over time [47]. This phenomenon appears to be exacerbated by the absence of additional curing. Furthermore, the potential for minor fiber clustering at higher volumes, acting as local defects, and increased void formation around fibers could also contribute to the strength reduction observed at 28 days. The resistance to this trend observed only in the SF60-75 mixture suggests that a sufficient quantity of well-distributed, high-aspect-ratio fibers is necessary to mitigate these detrimental effects significantly.

The data further reveal that the performance of polypropylene (PP) fiber mixes was consistently inferior to the control at both ages, with decreases ranging between 3% and 11% at 7 days and 25 and 40% at 28 days, which is consistent with their known material properties. The hydrophobic and smooth surface of PP fibers results in a weak interfacial bond with the hydrophilic geopolymer matrix, severely limiting stress transfer capacity and leading to premature pull-out upon cracking [2,17,18]. Furthermore, their low elastic modulus provides insufficient restraint against crack opening, resulting in a significant post-cracking strength drop [45,48]. The tendency for PP fibers to ball during mixing and their lower density, which increases the number of fibers per unit volume, can lead to non-uniform dispersion and the creation of more porous interfacial transition zones, further compromising the structural integrity. The slight mitigation of strength loss with higher PP fiber content is likely due to a marginal increase in crack-arresting mechanisms, though this effect is overwhelmingly negated by the aforementioned deficiencies.

**Figure 8:**
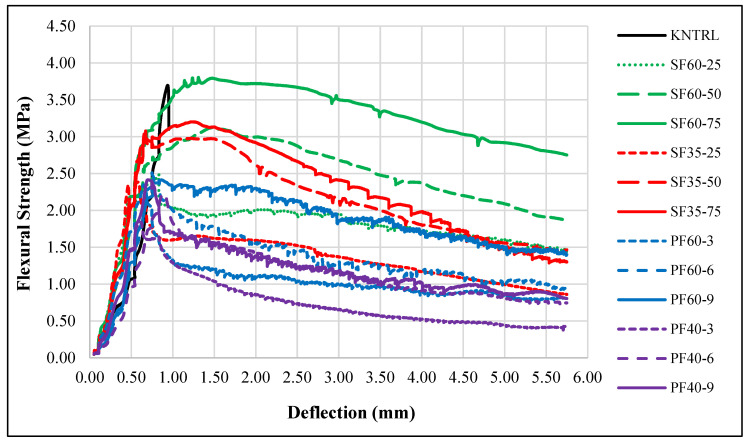
Flexural strength–displacement curves of fibrous geopolymer concretes. The different curves represent mixtures with varying fiber types, lengths, and dosages, as indicated in the legend.

**Figure 9:**
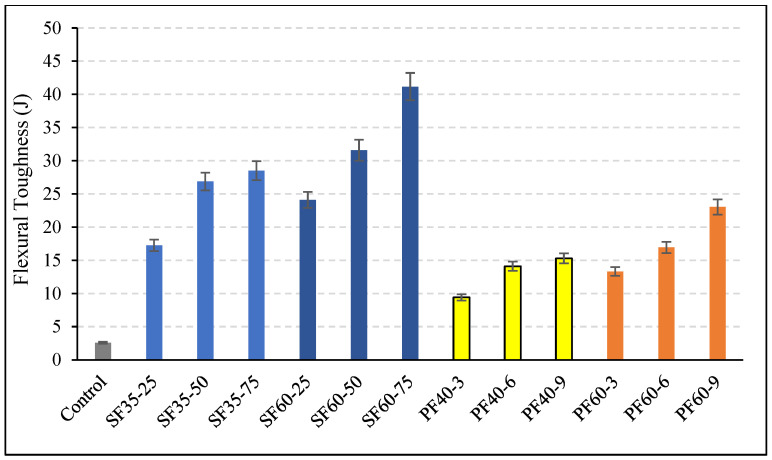
Flexural toughness values of fibrous geopolymer concretes. Different colors represent different mixture groups: gray indicates the control mixture, blue tones represent steel fiber (SF) mixtures, yellow tones represent polypropylene fiber mixtures with 40 mm length (PF40), and orange tones represent polypropylene fiber mixtures with 60 mm length (PF60).

Despite the reduction in ultimate flexural strength for most mixes at 28 days, the load–displacement curves (Figure 8) demonstrate that both fiber types fundamentally alter the failure mode from brittle to ductile. The calculated toughness values (Figure 9) show a remarkable increase, by a factor of 3–8 for PP fibers and 6–15 for steel fibers, which is a direct consequence of fiber bridging, enabling sustained stress redistribution and energy absorption after the first crack [2,16]. The superior toughness provided by steel fibers is a function of their higher modulus of elasticity, allowing them to absorb more energy and sustain higher stresses during pull-out [49]. The greater effectiveness of longer fibers in enhancing toughness, as seen in the SF60 series, can be explained by their larger surface area for bond development and higher resistance to being pulled out from the matrix [16].

The influence of fiber properties on crack-bridging mechanisms can also be interpreted within fracture mechanics frameworks developed for fiber-reinforced cementitious composites. In particular, the Updated Bridged Crack Model (UBCM) describes the post-cracking response of fiber-reinforced concrete by explicitly considering the bridging stresses generated by fibers across crack surfaces. The model has been successfully applied to both FRC and HRC members, demonstrating its ability to capture the influence of fiber volume fraction, fiber mechanical and geometrical properties, matrix characteristics, and structural size on the post-cracking behavior [50,51]. The UBCM predicts the energy dissipation during crack propagation as a function of the brittleness numbers, providing a rational framework for understanding how fiber parameters affect the energy absorption capacity of the composite. The model shows that the energy dissipation is maximized when the brittleness numbers are above their critical values, corresponding to a stable (ductile) post-cracking response [50,51,52].

In conclusion, the present findings demonstrate that while fibers are highly effective in enhancing ductility and toughness, their effect on the flexural strength of ambient-cured, slag-based geopolymer concrete is complex and contingent on fiber type and dosage. The results underscore that superior early-age performance does not guarantee efficacy. The strength reduction observed at 28 days highlights the critical importance of interfacial bond stability and matrix development under specific curing conditions, suggesting that optimized mix designs for fiber-reinforced geopolymers must account for these time-dependent phenomena to ensure durable performance. It can be concluded that the effectiveness of fiber reinforcement in geopolymers depends not only on initial bond strength but also on the stability of the fiber–matrix interface under specific curing regimes.

### 3.3. Compressive Strength

**Figure 10:**
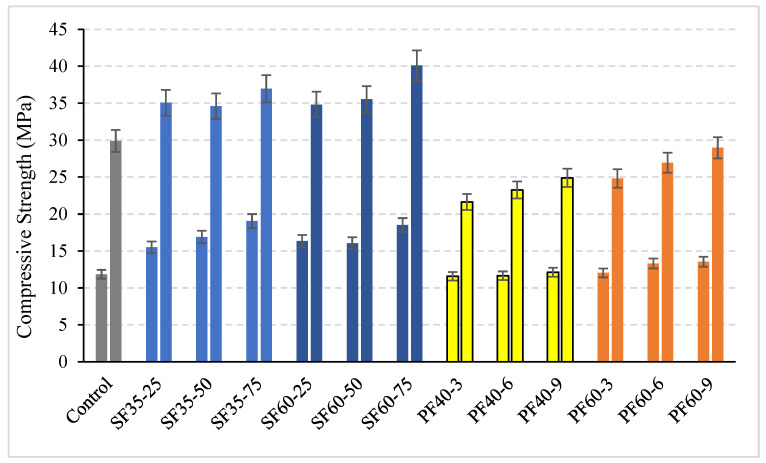
The 7- and 28-day compressive strengths of the mixtures. Different colors represent different mixture groups: gray indicates the control mixture, blue tones represent steel fiber (SF) mixtures, yellow tones represent polypropylene fiber mixtures with 40 mm length (PF40), and orange tones represent polypropylene fiber mixtures with 60 mm length (PF60).

The development of the compressive strength of all mixtures at 7 and 28 days is presented in Figure 10. The results indicate a clear positive influence of steel fiber reinforcement at both testing ages.

For instance, specimens incorporating 35 mm steel fiber demonstrated a substantial increase in 7-day compressive strength, with enhancements ranging from 30% to 60% as the fiber content increased from 25 to 75 kg/m^3^. Although this strengthening effect remained evident at 28 days, its magnitude was less pronounced. A positive correlation was observed between fiber content and compressive strength, while the influence of fiber length, though beneficial, was comparatively limited.

In contrast, the performance of PP fiber-reinforced mixtures was more complex and time-dependent. At 7 days, mixtures containing 40 mm PP fibers showed negligible change compared to the control, whereas those with 60 mm fibers exhibited strength gains of approximately 15%. However, a significant reversal was observed at 28 days, with all PP-fiber mixtures exhibiting lower compressive strengths than the control, showing reductions of up to 27%. A higher PP fiber content only slightly attenuated this strength loss, yet all values remained inferior to the plain geopolymer reference.

The beneficial effect of steel fibers on compressive strength is well-documented in the literature [1,2,53]. The hooked-end morphology and rough surface of the steel fibers facilitate mechanical interlocking and improve bonding with the geopolymer matrix [4]. Furthermore, higher fiber contents reduce the average spacing between fibers, which more effectively restricts the initiation and propagation of micro-cracks under compressive loads [1]. The fibers used in this study, being hooked at both ends, are consequently believed to enhance fiber–matrix adhesion significantly, leading to the observed strength increases.

PP fibers, however, operate through different and ultimately less effective mechanisms. The early-ages strength improvement seen with 60 mm fibers is likely due to their ability to bridge incipient shrinkage cracks, thereby reducing lateral expansion and delaying the formation of critical cracks [20,28]. At maturity, this initial benefit is superseded by the detrimental effects of poor fiber–matrix adhesion and the formation of voids and defects around the fibers. The inherently hydrophobic nature of PP fibers promotes water repulsion during mixing, leading to increased air bubble formation at the critical fiber–matrix interface [20,25,54]. This effect, compounded by potential fiber agglomeration, ultimately compromises the compressive strength [20,26].

These findings are consistent with microstructural evidence from the literature.

Electron microscope observations by Bellum et al. [2] confirmed that steel fibers achieve a superior bond with the geopolymer matrix to PP fibers. Similarly, Pham et al. [4] reported that hooked-end steel fibers yielded higher compressive strength and reduced deformations relative to PP fibers. The lower intrinsic tensile strength of PP fibers also contributes less to the overall confinement and mechanical performance of the composite under compression [1]. The results of this study, which show consistently higher compressive strengths in steel fiber mixtures, therefore support the established conclusion that steel fibers provide superior mechanical performance in geopolymer composites compared to polypropylene fibers.

## 4. Conclusions

This study presents a comprehensive experimental investigation into the effects of steel (SF) and polypropylene (PP) fibers on the workability, flexural strength, compressive strength, and toughness of slag-based geopolymer concrete. Based on the findings, the following conclusions can be drawn:

- Workability: The incorporation of fibers consistently reduced the workability of fresh geopolymer mixtures, as measured by the slump test. PP fibers had a more pronounced negative effect than steel fibers, reducing slump by up to ~45% compared to ~40% for steel fibers. The reduction was exacerbated by increasing both fiber length and dosage, with fiber content being the dominant factor.
- Flexural Strength: A significant time-dependent behavior was observed. Steel fibers significantly enhanced early-age (7-day) flexural strength, with gains of 20–38%. However, this positive effect drastically diminished at 28 days, where most steel fiber mixes underperformed compared to the control, with reductions of up to 20%. The SF60-75 mix was a notable exception, maintaining a 9% improvement. PP fibers reduced flexural strength at both improvements.
- Ductility and Toughness: Both fiber types remarkably improved the ductility and post-cracking behavior of the geopolymer concrete, transforming its failure mode from brittle to ductile. Steel fibers were substantially more effective, increasing flexural toughness by a factor of 6–15, compared to 3–8 for PP fibers. Fiber length was a more influential parameter on toughness than fiber content.
- Compressive Strength: Steel fibers reliably increased compressive strength at both 7 and 28 days. The 7 days strength of SF35 mixes increased by 30–60% with increasing fiber content. PP fibers provided a slight strength gain at 7 days (~15% for 60 mm fibers) but resulted in significant strength loss at 28 days, with reductions of up to 27%.

The overall mechanical performance of steel fibers was found to be superior to that of polypropylene fibers. This is attributed to their superior bond characteristics, a result of their hydrophilic nature, rough surface, and hooked ends, which enable effective stress transfer, crack bridging, and confinement. The negative performance of most fiber-reinforced mixes, particularly in flexural strength, is postulated to be a result of time-dependent degradation of the fiber–matrix interface under ambient curing conditions, highlighting the critical importance of the curing regime and interfacial stability for the durability of fiber-reinforced geopolymer composites.

## Figures and Tables

**Table 1:** Properties of blast furnace slag.

Component (%)	Blast Furnace Slag
SiO_2_	39.45
Fe_2_O_3_	1.03
Al_2_O_3_	13.00
CaO	31.14
MgO	7.67
Na_2_O	0.41
K_2_O	0.96
SO_3_	1.35
MnO	2.97

**Table 2:** Some physical and chemical properties of sodium silicate (Na_2_SiO_3_).

Property	Analysis Value
Appearance	Transparent, clear
Sodium Oxide—Na_2_O (%)	9.11
Silicon Dioxide—SiO_2_ (%)	27.78
Water—H_2_O (%)	63.11
Module (SiO_2_/Na_2_O)	3.05
Density (at 20 °C, g/cm^3^)	1.37

**Table 3:** Some physical and chemical properties of sodium hydroxide.

Property	Analysis Value
Appearance	White in color, flake shaped
Sodium Oxide—NaOH (%)	98.00
Sodium Chloride—NaCl (%)	0.10
Sodium Carbonate—Na_2_CO_3_ (%)	0.40
Density (at 20 °C, g/cm^3^)	2.13

**Table 4:** Some physical properties of aggregates.

Properties	0–5 mm	5–15 mm	15–25 mm
Loose Unit Volume Weight (kg/m^3^)	1584	1473	1458
Compact Unit Volume Weight (kg/m^3^)	1679	1560	1532
Dry Specific Gravity	2.64	2.65	2.70
Saturated Dry Surface Specific Gravity	2.68	2.70	2.71
Water Absorption (%)	1.67	0.88	0.23

**Table 5:** Sieve analysis results of aggregates.

Sieve Size (mm)	Passing (%)		
	0–5 mm	5–15 mm	15–25 mm
31.5	100	100	100
22.4	100	100	99.3
16	100	100	23.2
8	100	51	0
4	91.6	7.4	0
2	63.5	0	0
1	41.9	0	0
0.50	25.6	0	0
0.250	15	0	0
0.125	8.1	0	0

**Table 6:** Characteristic properties of steel and polypropylene fibers.

Property	ST60-67	ST35-64	PP60	PP40
Density (g/cm^3^)	7.85	7.85	0.91	0.91
Length (mm)	60	35	60	40
Diameter (mm)	0.90	0.55	0.95	0.72
Aspect Ratio (Length/Diameter)	67	64	63	56
Elasticity Moduli (GPa)	210	210	7.3	8.5
Tensile Stress (MPa)	1160	1345	510	550
Melting Point (°C)	>1500	>1500	160	160

**Table 7:** Mixing proportion (kg/m^3^) and curing conditions of geopolymer concretes.

Component	Control	SF60-25	SF60-50	SF60-75	SF35-25	SF35-50	SF35-75	PF60-3	PF60-6	PF60-9	PF40-3	PF40-6	PF40-9
Blast Furnace Slag	300	300	300	300	300	300	300	300	300	300	300	300	300
Sodium Hydroxide	20.9	20.9	20.9	20.9	20.9	20.9	20.9	20.9	20.9	20.9	20.9	20.9	20.9
Sodium Silicate	151.2	151.2	151.2	151.2	151.2	151.2	151.2	151.2	151.2	151.2	151.2	151.2	151.2
Additional Water	69.6	69.6	69.6	69.6	69.6	69.6	69.6	69.6	69.6	69.6	69.6	69.6	69.6
0–5 mm Fine aggregate	1047.9	1043.0	1038.1	1033.2	1043.0	1038.1	1033.2	1042.8	1037.8	1032.7	1042.8	1037.8	1032.7
5–15 mm Coarse aggregate	325.2	323.7	322.2	320.7	323.7	322.2	320.7	323.6	322.0	320.5	323.6	322.0	320.5
15–25 mm Coarse aggregate	433.6	431.6	429.6	427.5	431.6	429.6	427.5	431.5	429.4	427.3	431.5	429.4	427.3
ST60-67	0	25	50	75	0	0	0	0	0	0	0	0	0
ST35-64	0	0	0	0	25	50	75	0	0	0	0	0	0
PP60	0	0	0	0	0	0	0	3	6	9	0	0	0
PP40	0	0	0	0	0	0	0	0	0	0	3	6	9
TOTAL	2386	2402	2419	2435	2402	2419	2435	2380	2375	2368	2380	2375	2368
Water/binder ratio *	0.55	0.55	0.55	0.55	0.55	0.55	0.55	0.55	0.55	0.55	0.55	0.55	0.55
Na_2_O Ratio (%)	10	10	10	10	10	10	10	10	10	10	10	10	10
Ms Ratio	1.40	1.40	1.40	1.40	1.40	1.40	1.40	1.40	1.40	1.40	1.40	1.40	1.40
Curing Condition	25 °C air	25 °C air	25 °C air	25 °C air	25 °C air	25 °C air	25 °C air	25 °C air	25 °C air	25 °C air	25 °C air	25 °C air	25 °C air

* Additional water and water from sodium silicate were taken into account in the calculation of the water/binder ratio.

**Table 8:** Slump values of geopolymer mixtures.

Mixtures	Slump (cm)
Control	25
SF60-25	24
SF60-50	20
SF60-75	15
SF35-25	24
SF35-50	22
SF35-75	19
PF60-3	20
PF60-6	17
PF60-9	14
PF40-3	24
PF40-6	20
PF40-9	15

## Data Availability

The original contributions presented in this study are included in the article. Further inquiries can be directed to the corresponding author.
